# Spatiotemporal mathematical modelling of mutations of the *dhps* gene in African *Plasmodium falciparum*

**DOI:** 10.1186/1475-2875-12-249

**Published:** 2013-07-17

**Authors:** Jennifer A Flegg, Anand P Patil, Meera Venkatesan, Cally Roper, Inbarani Naidoo, Simon I Hay, Carol Hopkins Sibley, Philippe J Guerin

**Affiliations:** 1 WorldWide Antimalarial Resistance Network (WWARN), University of Oxford, Oxford, UK; 2 Centre for Tropical Medicine, Nuffield Department of Clinical Medicine, University of Oxford, Oxford, UK; 3 Department of Zoology, Spatial Ecology and Epidemiology Group, University of Oxford, Oxford, UK; 4 Howard Hughes Medical Institute, University of Maryland School of Medicine, Baltimore, MD, USA; 5 WorldWide Antimalarial Resistance Network Molecular Module, University of Maryland School of Medicine, Baltimore, MD, USA; 6 London School of Hygiene and Tropical Medicine, Keppel Street, London WC1E 7HT, UK; 7 Malaria Research Unit, Medical Research Council, Durban, South Africa; 8 WorldWide Antimalarial Resistance Network (WWARN) and Department of Genome Sciences, University of Washington, Seattle, USA

## Abstract

**Background:**

*Plasmodium falciparum* has repeatedly evolved resistance to first-line anti-malarial drugs, thwarting efforts to control and eliminate the disease and in some period of time this contributed largely to an increase in mortality. Here a mathematical model was developed to map the spatiotemporal trends in the distribution of mutations in the *P. falciparum* dihydropteroate synthetase (*dhps*) gene that confer resistance to the anti-malarial sulphadoxine, and are a useful marker for the combination of alleles in *dhfr* and *dhps* that is highly correlated with resistance to sulphadoxine-pyrimethamine (SP). The aim of this study was to present a proof of concept for spatiotemporal modelling of trends in anti-malarial drug resistance that can be applied to monitor trends in resistance to components of artemisinin combination therapy (ACT) or other anti-malarials, as they emerge or spread.

**Methods:**

Prevalence measurements of single nucleotide polymorphisms in three codon positions of the dihydropteroate synthetase (*dhps*) gene from published studies of *dhps* mutations across Africa were used. A model-based geostatistics approach was adopted to create predictive surfaces of the *dhps*540E mutation over the spatial domain of sub-Saharan Africa from 1990-2010. The statistical model was implemented within a Bayesian framework and hence quantified the associated uncertainty of the prediction of the prevalence of the *dhps*540E mutation in sub-Saharan Africa.

**Conclusions:**

The maps presented visualize the changing prevalence of the *dhps*540E mutation in sub-Saharan Africa. These allow prediction of space-time trends in the parasite resistance to SP, and provide probability distributions of resistance prevalence in places where no data are available as well as insight on the spread of resistance in a way that the data alone do not allow. The results of this work will be extended to design optimal sampling strategies for the future molecular surveillance of resistance, providing a proof of concept for similar techniques to design optimal strategies to monitor resistance to ACT.

## Background

Despite increased global efforts to control malaria, *Plasmodium falciparum* malaria remains a major public health issue [1], and a priority of the Millennium Development Goals. Attempts to eliminate malaria in Africa have been hampered by resistance of *P. falciparum*, first to chloroquine and later to sulphadoxine-pyrimethamine (SP). The recent emergence in Southeast Asia of resistance to artemisinin, a key component of current first-line therapy, is a major worldwide public health concern [2,3], and a significant impediment to future malaria eradication [4].

Chloroquine was the most common first-line therapy until the late 1990s. Resistance to chloroquine was first detected in Asia in the 1950s [5,6] and spread to Africa in the late 1970s [7]. As chloroquine failure became widespread throughout sub-Saharan Africa in the following decades, it was replaced with SP in some countries as the first-line therapy. Resistance to SP rapidly followed its introduction [8] and has been extensively documented in many regions of Africa [9].

The emergence and spread of parasite resistance is a two-staged process: *de novo* appearance of a parasite genotype that confers better survival in the presence of the drug, followed by the preferential transmission of organisms with the acquired resistance [10]. Molecular markers alone cannot be used to predict treatment outcomes in individual patients, because other factors such as immunity, nutritional status, haemoglobinopathies and variation in drug absorption and metabolism can also affect clinical outcomes [11-14]. However, validated molecular markers are useful tools for mapping and monitoring anti-malarial resistance at a population level and as a surveillance tool, to indicate an aggregated measure of increased risk for clinical failure [15].

Point mutations occur *de novo*, independently within individual parasites, but resistance to SP is a complex trait, requiring a specific constellation of changes in two unlinked genes. Molecular studies have shown that the combination of the three mutations in *dhfr* (S108N, C59R, N51I) defines a key highly pyrimethamine-resistant combination or haplotype. A parasite that also carries a ‘double’ mutant allele of *dhps* (A437G, K540E) is strongly associated with increased risk of SP treatment failure in Africa [16-19].

These molecular markers have been productively used in population analysis of the molecular changes underlying the development of SP resistance in Africa. The correct set of point mutations in the *dhps* and *dhfr* genes occur together relatively infrequently but once assembled, parasites that carry these combinations then spread over large-scale geographic regions. In fact, the emergence of parasites with mutations in the *dhps* gene, is almost always observed in populations that already carry the triple mutant allele of *dhfr *[8]. Most commonly in Africa, mutant alleles of *dhps* are selected in a stepwise fashion; an intermediate allele that carries A437G alone, followed by selection of the A437G + K540E associated with clinical SP resistance. Maps of the distributions of observed *dhps* mutant haplotypes show that the 437G single mutant haplotype is commonly found in West Africa while the 437G + 540E double mutant haplotype is prevalent throughout East Africa [20]. The study further analysed the number of independent origins of these alleles and showed that the dispersal of five major mutant lineages (three different 437G alleles and two 437G + 540E double mutants) accounted for the majority of the observed haplotypes in African populations. It is worth noting again that mutations in the *dhps* gene only arise in populations that carry a high prevalence of the triple *dhfr* mutation [8]. The addition of A581G and/or A613T/S confers even higher levels of resistance *in vitro*[21]. Although comparatively rare, the 581G in combination with 437G and 540E in the form of a triple mutant allele is responsible for a measureable deterioration in SP efficacy [22,23]. Currently the WHO recommends SP for intermittent prophylactic treatment of pregnant women and infants, but in populations where 50% or more of the parasites carry a *dhps*540E allele, this is no longer recommended [24,25].

Lessons learned from the spread of these markers of SP resistance in Africa can be used as a model of anti-malarial spread in the continent. This work was motivated, not solely to investigate the spatiotemporal spread of molecular markers that confer resistance to SP, but also to predict the likely spread of resistance to artemisinin combination therapy (ACT) when and where it arises.

## Methods

For each of the A437G, K540E and A581G mutations, the literature was searched and data extracted on the time and location of the survey, the number of people who were tested and the number of people who were positive for that mutation: that information was recorded in a database [9,25]. For visualizations of the molecular marker data, see [26] and [27]. Additional file 1 contains a summary of the timing and location of the surveys captured. In this paper the aim was to use the A437G, K540E and A581G data to infer a continuous surface for the prevalence of K540E, in order to inform an understanding of the emergence and dispersal of drug resistance in African populations of of *P. falciparum*. A continuous surface was inferred based upon the observations of prevalence, and a statistical approach employed to estimate the prevalence of *dhps*540E at locations between the observations.

Throughout this paper, the prevalence of *dhps*540E refers to the proportion of infected individuals in a population that are infected with one or more resistant clones. The distinction between the prevalence and frequency of a molecular marker is an important one. Briefly, the frequency of a molecular marker is the proportion of parasites in the population that carry the marker in question (taking into consideration that a single person can be infected with multiple clones) while the prevalence is the proportion of all individuals that are infected with one or more resistant clones. Even if the frequency of the molecular marker is the same across space and/or time, individuals will tend to be infected with more clones in regions of high malaria transmission. For this reason, frequency is the measure upon which the genetics of allele spread should ideally be modelled. However, genetic studies typically measure or report marker prevalence and while it is possible to use a statistical model to infer marker frequency from prevalence data, for the purposes of this paper the primary, individual patient level data were not available. Only a single aggregate prevalence from each study site and location was available.

The purpose of the modelling approach used in this paper was to generate a continuous surface, in both space and time, which approximates the prevalence of the *dhps*540E marker. That is, the *dhps* data, available only at discrete study locations and times, were used to predict the changing prevalence of *dhps*540E across the entire African continent from 1990-2010, thus providing insight on the spread of resistance, in both space and time, in a way that observations from the discrete data points alone can not provide. The model included estimates of the *P. falciparum* transmission intensity in 2010, as estimated by the spatiotemporal models developed by the Malaria Atlas Project [1]. Since multiplicity of infection (MOI) is an indicator of, and positively correlated with, transmission intensity, the inclusion of transmission compensates, to some extent, for the omission of MOI. Full details of the model are provided in Additional file 2.

There were two main stages to the statistical methodology for the spatiotemporal prediction of the *dhps*540E molecular marker prevalence, which are outlined briefly here (see Additional file 2 for details). Firstly, based on the observed data, the model parameters were estimated. Secondly, given the model parameters in the first stage, *dhps*540E prevalence was predicted on a 25 × 25 km grid of sub-Saharan Africa in each year from 1990 to 2010. For each location, a distribution of prevalences were drawn from the model and summarized using the median statistic to create a single continuous surface. The standard deviation surface is also presented alongside the median maps as a summary of the associated uncertainty in the predictions at each location.

The model validity was assessed to ensure that the interpretation of the model output was valid. The *dhps*540E dataset was divided into five groups, at random; each subset was treated as a validation set to test the model’s predictive ability. For each of the five subsets of data, the model was run with one dataset withheld and the ability of the model to predict that subset was tested against the actual withheld data. The predictive results for each of the five subsets of data were pooled, so that each *dhps*540E observation had an associated predictive validation value. Further details about the validation procedures are given in Additional file 3.

## Results

### *dhps*540E marker prevalence predictive maps

To generate predictive maps, all of the available data (from all years) were used to inform a single spatiotemporal model. From the results of this spatiotemporal model, predictive maps for the years 1990, 2000 and 2010 were generated.

### Spatial maps in 1990

**Figure 1:**
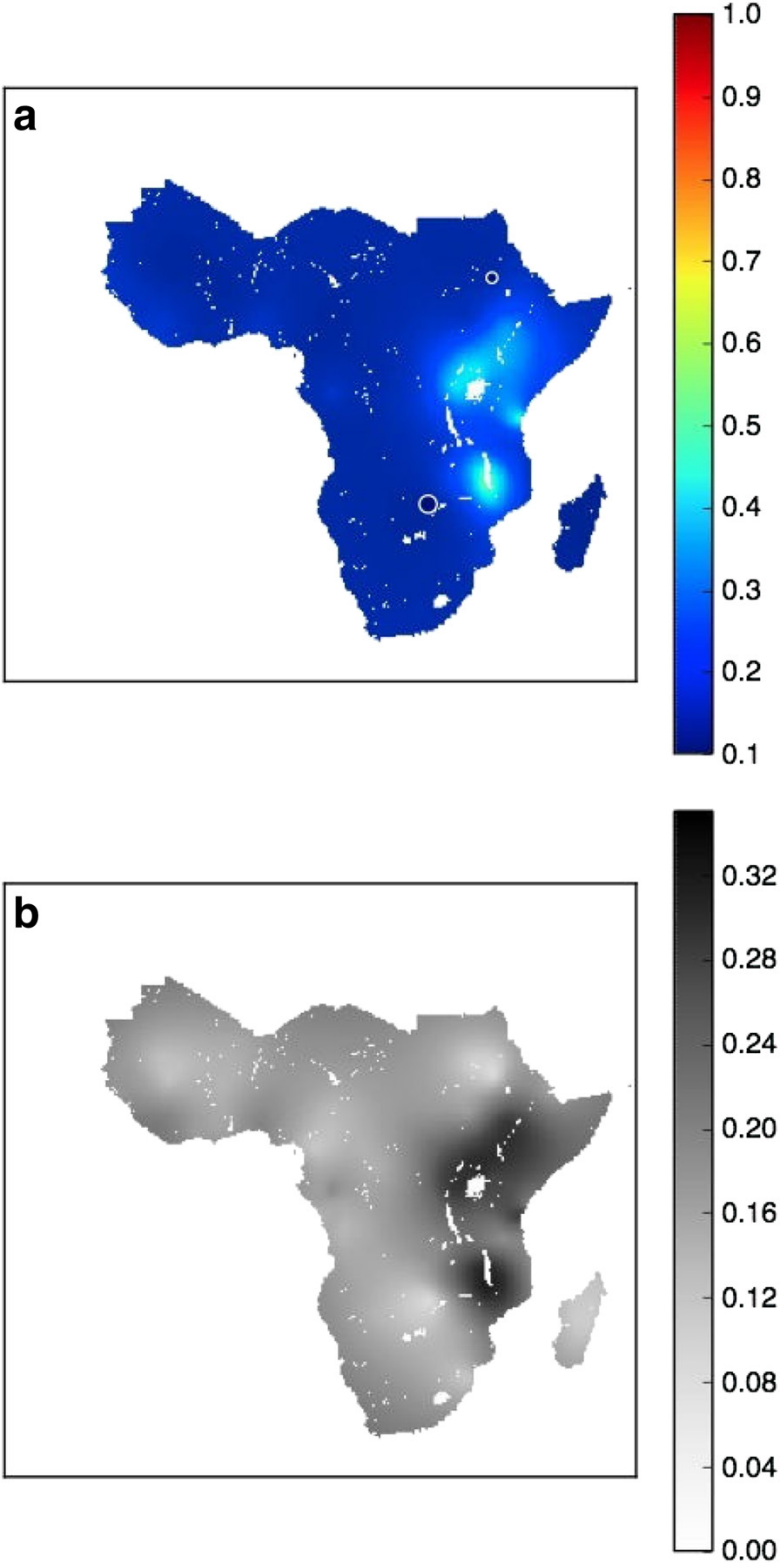
**Spatial maps in 1990.** The spatial distribution of median *dhps*540E *prevalence* from the model output in 1990 **(a)** and the associated model uncertainty **(b)**. The median values of the predictive prevalence are represented as a continuous surface, with dark blue corresponding to low prevalences and dark red to high prevalences. Studies that were conducted before or during the year of 1990 are represented on the median surface maps by circles – where the colour of the circle is proportional to the observed *dhps*540E prevalence at that study location and the radius of the circle is proportional to the sample size of the study. All of the available data, from all years, were used to inform the 1990 map in the spatiotemporal model.

The predicted median *dhps*540E prevalence in sub-Saharan Africa in 1990 is presented in Figure 1(a), visualized as a continuous surface. A predicted *dhps*540E prevalence of 0.2 at a certain location in space and time means that of 100 individuals who carry *P. falciparum* at that time and location, 20 are predicted to carry parasites positive for the *dhps*540E marker. In 1990, the model predictions suggest that *dhps*540E prevalence was low across most of the continent. However, there were several regions in East Africa where the *dhps*540E prevalence was relatively high (see the light blue areas in Figure 1(a)). In 1990, only 7% of the sub-Saharan area had a *dhps*540E prevalence of more than 0.2.

Uncertainty maps are presented alongside the median maps as a summary of the associated uncertainty in the model predictions. Figure 1(b) illustrates the associated uncertainty in the model predictions for 1990. Uncertainty in the model predictions was measured as the standard deviation of the *dhps*540E predicted prevalences (see the Methods and Additional file 2 for more details). The uncertainty map for 1990 (Figure 1(b)) indicates high model confidence in predictions where the prevalence was low, while there are regions of relatively high uncertainty in East Africa (corresponding to the regions where the *dhps*540E prevalence was higher). While the uncertainty in 1990 may seem low, given the map and data presented in Figure 1(a), it is important to recall that all of the available data (from all years) were used to inform a single spatiotemporal model. The maps for 1990 (and subsequent years) were generated based on the results of this spatiotemporal model.

### Spatial maps in 2000

**Figure 2:**
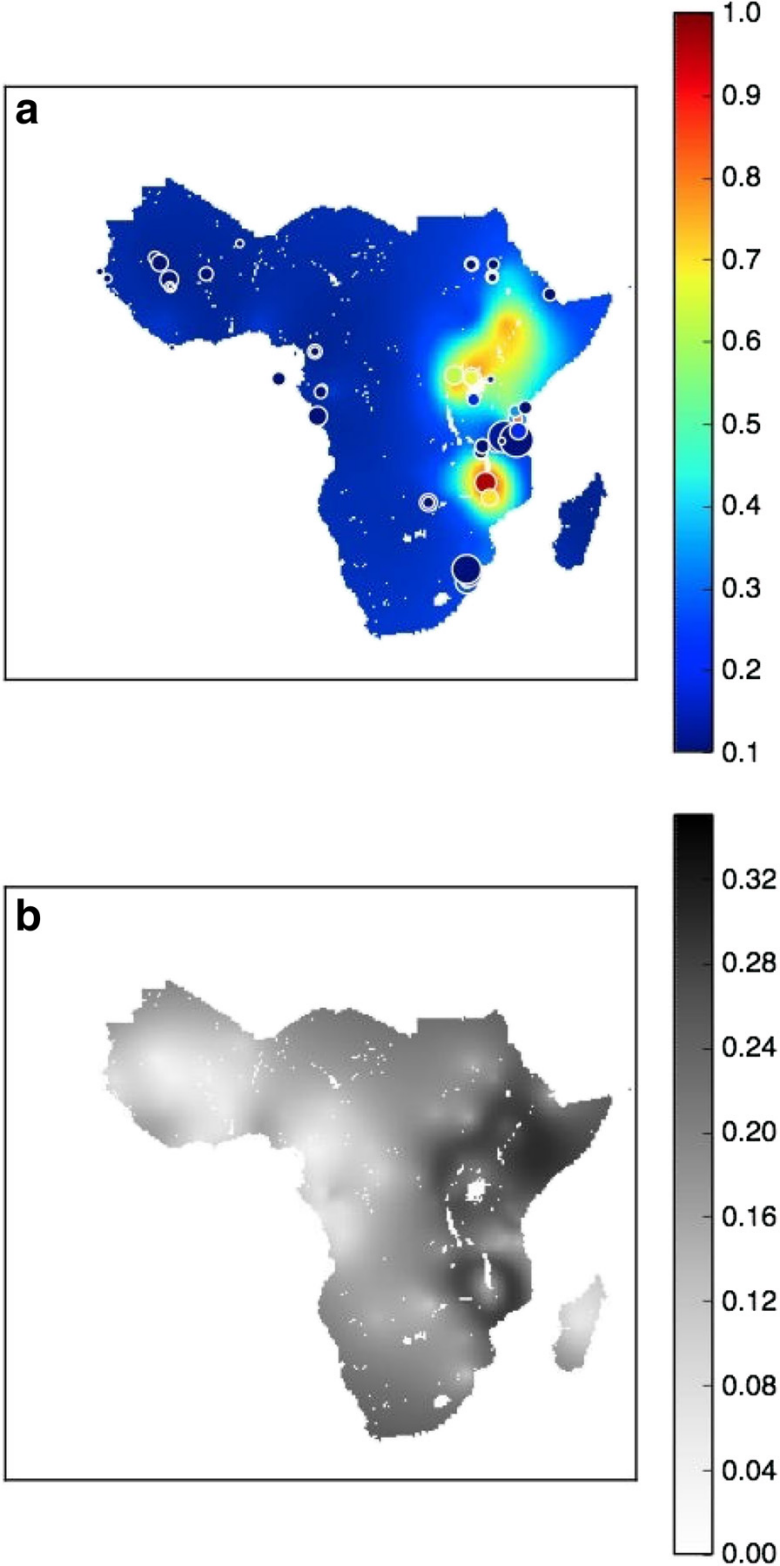
**Spatial maps in 2000.** The spatial distribution of median *dhps*540E *prevalence* from the model output in 2000 **(a)** and the associated model uncertainty **(b)**. Studies that were conducted before or during the year of 2000 are represented on the median surface maps. All of the available data, from all years, were used to inform the 2000 map in the spatiotemporal model.

Figure 2(a) and 2(b) illustrate the predictive median surface and the associated uncertainty, respectively, in 2000. In the interval between the 1990 and 2000 maps (see Figure 1(a) and 2(a)) the prevalence of *dhps*540E was predicted to have increased in East Africa, however the *dhps*540E prevalence in West Africa and Madagascar remained very low. In 2000, 18% of the area in sub-Saharan Africa had a *dhps*540E prevalence of more than 0.2, compared to only 7% in 1990. Figure 2(b) shows that the uncertainty in the 2000 model predictions has, overall, remained similar to the uncertainty map in 1990. The uncertainty map illustrates the highest uncertainty in regions where the predicted prevalence was moderate, which is consistent with the variance imposed under the binomial assumption in the model.

### Spatial maps in 2010

**Figure 3:**
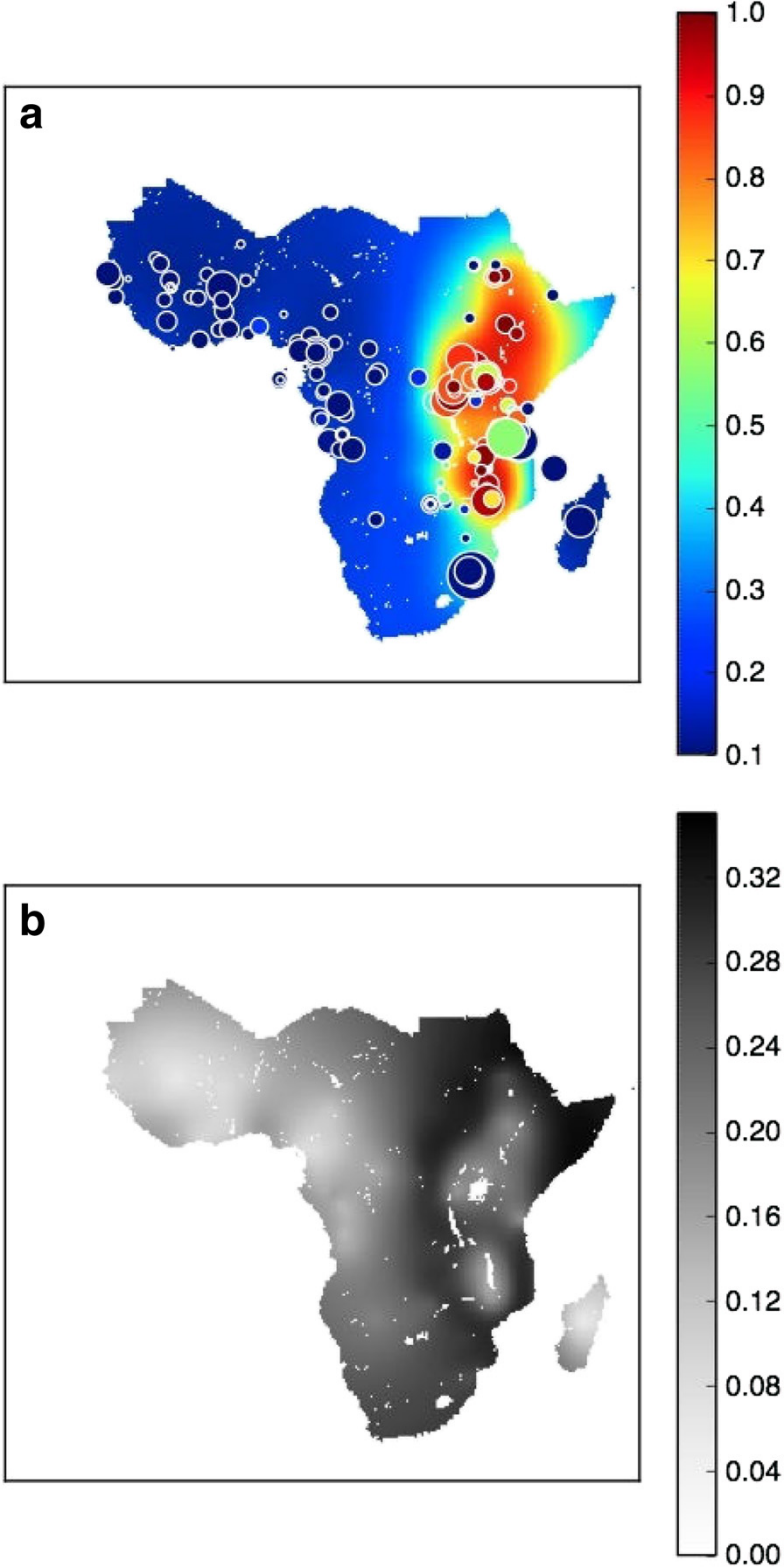
**Spatial maps in 2010.** The spatial distribution of median *dhps*540E *prevalence* from the model output in 2010 **(a)** and the associated model uncertainty **(b)**. Studies that were conducted before or during the year of 2010 are represented on the median surface maps. All of the available data, from all years, were used to inform the 2010 map in the spatiotemporal model.

Figure 3(a) and 3(b) show the *dhps*540E predicted prevalence and uncertainty for 2010. Further spatial spread of the *dhps5*40E marker was predicted to have occurred since the 2000 map (see Figure 2(a)). An increased proportion of sub-Saharan land area, 38%, was predicted to have a *dhps*540E prevalence above 0.2. East Africa was predicted to have predominantly high levels of *dhps*540E prevalence, while West Africa continues with relatively low prevalence. The uncertainty map (Figure 3(b)) indicates an increase in uncertainty in central and eastern Africa.

An animation of the predictive median surfaces over the years 1990-2010 (https://www.wwarn.org/about-us/news/spread-antimalarial-molecular-resistance-sulphadoxine-pyrimethamine) clearly captures the spatiotemporal changes in the predicted *dhps*540E distributions, showing the emergence of regions of high *dhps*540E prevalence in East Africa and the predicted spread of the *dhps*540E marker from these regions.

### Interpretation of uncertainty

**Figure 4:**
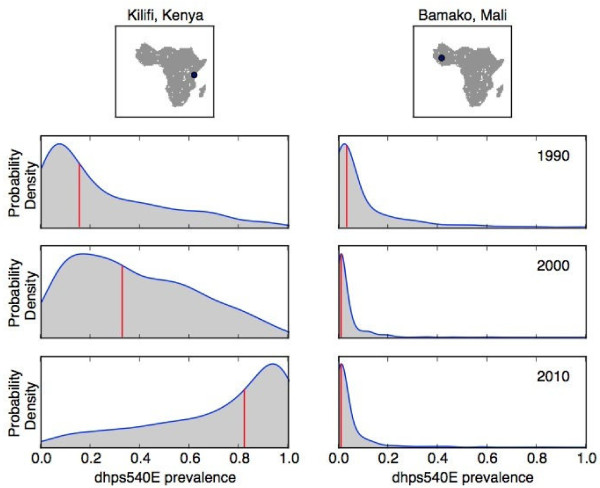
**Predictive distributions of *dhps*540E prevalence at two locations.** The *dhps*540E prevalence distribution near Kilifi, Kenya in East Africa (left hand side panels) and near Bamako, Mali in West Africa (right hand side panels). The rows relate to the predictive distribution in 1990, 2000 and 2010, respectively. The vertical red lines represent the median *dhps*540E prevalence of the distributions.

To help interpret the uncertainty maps, the individual distributions for two locations in 1990, 2000 and 2010 are visualized (see Figure 4). The two locations were chosen to represent a location where both the prevalence of *dhps5*40E and the uncertainty were low, in West Africa (the Bamako region, Mali), and a location where *dhps*540E prevalence increased from 1990-2010 and the uncertainty was relatively high, in East Africa (the Kilifi region, Kenya). It should be noted that using the methodology outlined in this paper, it is not possible to discuss the “true” *dhps*540E prevalence in any location (including the two example regions considered here). Instead, the prevalence can only be described in a distributional sense or summarized using statistics.

The median predicted *dhps*540E prevalence in the Bamako region did not change significantly over the time period: the median (interquartile range (IQR)) prevalence in 1990, 2000 and 2010 was 3% (1-10)%, 1% (0-4)% and 1% (0-4)%, respectively. The low predicted prevalence in the Bamako region in 1990, 2000 and 2010 can be seen in Figure 1(a), 2(a), and 3(a), in the dark blue colour of the maps in West Africa. The uncertainty in the Bamako region remained low over time: the standard deviation of the prevalence was 0.14, 0.06 and 0.08 in 1990, 2000 and 2010, respectively. The higher uncertainty in 1990 compared to 2000 and 2010 can be seen in the probability distributions on the right hand side of Figure 4: the distribution (blue curve) in 1990 is wider than the subsequent years and hence there is higher uncertainty in the prevalence. Furthermore, the wider IQR of prevalence in the Bamako region in 1990 (1-10%) compared to 2000 (0-4%) and 2010 (0-4%) is also representative of larger uncertainty. However, the probability distributions in the Bamako region are generally quite narrow (right hand side of Figure 4), indicative of low uncertainty. The low uncertainty in the Bamako region is visualized in Figure 1(b), 2(b), and 3(b) by the lighter gray colour of the maps in West Africa.

In the Kilifi region, the predicted median *dhps*540E prevalence increased significantly over time: 15%, 33% and 82% in 1990, 2010 and 2010, respectively. The increase in prevalence during this period in the Kilifi region can be seen in Figure 1(a), 2(a), and 3(a) by the rising prevalence in East Africa. The associated uncertainty in the Kilifi region was much higher than the Bamako region: in 1990, 2000 and 2010 the standard deviation was 0.24, 0.24 and 0.27, respectively. The probability distributions for the Kilifi region (left hand side of Figure 4) were therefore considerably wider than for the Bamako region, as are the IQR for prevalence (1990: 6-39%, 2000: 16-55%, 2010: 54-95%). In Figure 1(b), 2(b), and 3(b), the higher level of uncertainty in the predictions in the Kilifi region is indicated by the darker gray colour in regions of East Africa. Locations in 2010 with large amounts of uncertainty in the predicted *dhps*540E prevalence (see Figure 3(b)) may benefit from further molecular surveillance within the area since the uncertainty might be reduced.

### Temporal trends

**Figure 5:**
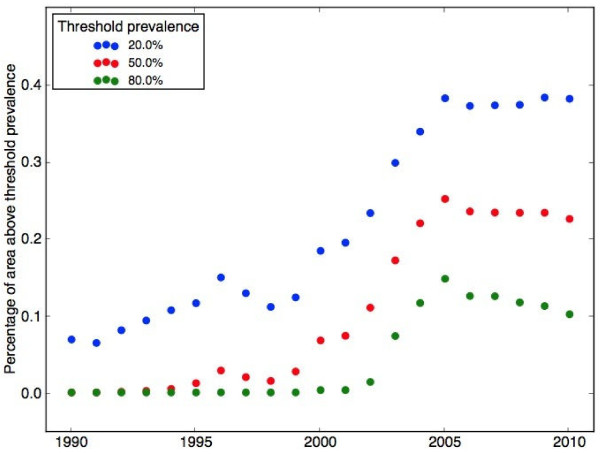
**Temporal trends in proportion of sub-Saharan Africa with *dhps*540E prevalence exceeding threshold.** The proportion of sub-Saharan Africa with a predictive median *dhps*540E prevalence exceeding 0.2 (blue), 0.5 (red) and 0.8 (green) over the period 1990-2010.

Figure 5 shows the proportion of sub-Saharan Africa under consideration within the model with a predictive *dhps*540E median exceeding 0.2 (blue), 0.5 (red) and 0.8 (green) over the years 1990 to 2010. There is a clear trend of an increasing area with a *dhps*540E prevalence exceeding 0.2 (blue dots) from 1990 to 2005. After 2005, the proportion of area with a prevalence of more than 0.2 stabilized at just below 40%. The period of time from 1998 to 2005 shows the fastest increase in the proportion of area with a prevalence of more than 0.2 and 0.5 (red dots), while high prevalence locations (exceeding 0.8 – green dots) were predicted to have started around 2002 and increased rapidly until 2005.

### Validation statistics

The validity of the model was assessed by rerunning the model five times, each time with a subset of 80% of the complete *dhps*540E dataset. The results were then pooled, so that each *dhps*540E observation had an associated predictive validation value, from a model where this observation was not included. The mean error in the generation of *dhps*540E prevalence estimates at the 238 data locations was 1.83%, indicating that overall bias was small. The mean absolute error was found to be 12.9%, while the correlation co-efficient between the observed *dhps*540E prevalences and their associated validation values was 0.86, demonstrating a very strong linear agreement between the observed and predicted prevalences. Full results of the validation procedure can be found in Additional file 3.

## Discussion

In this paper a spatiotemporal model was developed for the prevalence of the *dhps*540E marker in sub-Saharan Africa. Continuous maps in space and time were presented for the median predicted *dhps*540E prevalence, along with the associated uncertainty in these predictions. Based on the data available, the emergence and spread of the *dhps*540E mutation has been visualized at all space-time points within a 25 x 25 km gridded sub-Saharan Africa domain for each year between 1990 and 2010. In modelling the spatiotemporal emergence and spread of drug resistance in Africa, the results of this work have significance for public health and the future management of artemisinin resistance in Africa.

The maps presented here (see Figures 1, 2 and 3) visualize the changing prevalence of the *dhps*540E mutation in sub-Saharan Africa and are a useful tool to inform public health policy on the continuing use of SP. It could be used to advise on regions where SP should not be used as a partner drug, as a seasonal malarial chemoprevention or as an intermittent preventative treatment. For example, currently the WHO recommends SP for intermittent prophylactic treatment of pregnant women and infants, but in populations where 50% or more of the parasites carry a *dhps*540E allele, this approach is no longer recommended [24,25]. These regions could easily be predicted using the model developed here.

Previous attempts to spatially map the molecular markers associated with SP have been made [28], however the model presented here extends this work since it is spatiotemporal and also because it is embedded in a Bayesian framework, allowing the uncertainty to be quantified. The predicted surface generated as part of this previous work differs considerably from the maps of *dhps*540E prevalence presented here because the previous study considers all the prevalence data to influence a single continuous map equally, regardless of the year in which the data were collected. Whereas here a space-time model framework was considered that ensured that studies conducted in a particular year influence the map for that year more than a study conducted many years before.

The limited number of spatial and/or temporal data points available in certain regions of Africa, greatly affect the predictive value of the model, and as a result, the level of uncertainty of the median *dhps*540E prevalence estimates can be relatively high (see Figure 3). This observation in itself is important. While SP has been the most studied anti-malarial, in terms of molecular markers over the last three decades, major gaps of information remain. These gaps reflect the absence of research activities in particular regions, a lack of systematic reporting of available data, and/or a limited access to unpublished data.

There are several ways that this work could be extended: in addition to transmission intensity, which was incorporated into the current model, other, informative covariates, such as human population estimates within Africa and spatial environmental variables could be included within the model framework. The temporal trends of *dhps*540E at certain locations (see Figure 4 for the predictive distributions of *dhps*540E prevalence in the Kilifi region, Kenya and the Bamako region, Mali in 1990, 2000 and 2010) are likely to be dependent both on the national anti-malarial policy and actual drug use. Likewise, the temporal trends shown in Figure 5 (illustrating the proportion of sub-Saharan Africa with a predictive median *dhps*540E prevalence exceeding various thresholds) will be related to both factors. For instance, the period of rapid increase in the temporal trends shown in Figure 5 corresponds to the years when the highest number of African countries were recommending SP as a first-line therapy [29]. An extension of this work will add SP drug pressure and national anti-malarial drug policy to inform more accurately the spatiotemporal spread of SP resistance.

Many malaria endemic countries are working to eliminate malaria and up-to-date intelligence on the various parameters that are likely to impede such progress is critically important. The gaps in data reflect not only technical, human, political and financial constraints but also difficulties in establishing the optimal sites to survey, in terms of predictive value and representativeness. The work presented here will be extended to investigate the design of future surveillance, informed by the level of uncertainty, the level of *dhps*540E prevalence, malaria transmission [1] and human population density [30]. The aim would be to define the minimum spatiotemporal set of data necessary to design comprehensive surveillance matrices, allowing resistance mapping with acceptable level of uncertainties. This concept, called “smart surveillance”, has the potential to inform a guided surveillance plan, less based on current research capacities and more on where informative data are most needed. By adopting such an approach, mathematical modelling can facilitate the information systems needed to optimize the current efforts in malaria elimination and eradication.

The methodology outlined in this paper serves as a proof of concept for the application of geospatial modelling techniques to other anti-malarial drugs, as well as forms of data other than molecular markers, and will allow anti-malarial resistance to ACT or novel drugs to be monitored in space and time.

## Competing interests

The authors declare that they have no competing interests.

## Authors’ contributions

SH, CR and PG designed the study. JF implemented the algorithms, with the help of AP. JF wrote the first draft of the manuscript. CR and IN supplied the molecular data. CS and MV helped with the interpretation of the results. All authors read and approved the final manuscript.

## Supplementary Material

Additional file 1 **A database of resistant *dhps *in African *P. falciparum *malaria.** Click here for file

Additional file 2 **Model based geostatistical framework for generating maps of *dhps*540E prevalence.** Click here for file

Additional file 3 Model validation procedures and results. Click here for file
