# Supplementary material for: Spatiotemporal mathematical modelling of mutations of the dhps gene in African Plasmodium falciparum
Source: Malar J. 2013 Jul 17;12:249. doi: 10.1186/1475-2875-12-249 (PMC3728261; doi:10.1186/1475-2875-12-249)
Supplement: Additional file 1 — A database of resistant dhps in African P. falciparum malaria. [file 1475-2875-12-249-S1.pdf]

Additional file 1. A database of resistant *dhps* in African *P. falciparum* malaria

A1.1 Assembling the *dhps* marker databases

Search strategies

Online literature searches for the terms "malaria" and "*dhps*" in studies published up until February 2011 were conducted using the Pubmed, African Journal Online and Bioline databases.

Inclusion criteria

Criteria for inclusion were published studies of *dhps* genotypes in *P. falciparum* infections in Africa, excluding studies of malaria imported to non-African countries and studies for which the sampling year could not be determined.

Data extraction and data entry

The full text of all studies meeting the inclusion criteria was reviewed to extract the number of samples tested and number positive for *dhfr* and *dhps* mutations. Data were recorded on a standard proforma containing predesigned data fields, including geo-referenced study site and study year, and subsequently entered into a relational database. Interactive online maps for each marker are provided at www.drugresistancemaps.org and http://www.wwarn.org/surveyor/.

A1.2 Data used for modelling

Prevalences of all *dhps*437G, 540E and 581G markers were used to inform the *dhps*540E prevalence maps. A summary of the data used for the modelling is presented in Table A1.1 Each study was referenced spatially by the longitude and latitude of the study site and temporally by the mid-point year of the study.

The spatial location of *dhps* marker data is shown in the left hand panels of Figure

A1.1. In these spatial plots, each data point is represented by a circle on the map, where the colour represents the observed prevalence of the marker at that location and the size of the circle is proportional to the sample size of the study. The right hand panel in Figure A1.1 illustrates the number of surveys conducted for each marker in each year (from 1987 to 2008) for the *dhps* markers.

Table A1.1 Summary of the *dhps* marker data. Number of studies in the database, the median observed prevalence of the marker, the median year of study and the median study sample size.

Marker	Number of studies	Median prevalence	Median year	Median sample size
*dhps*437G	229	0.57	2003	76
*dhps*540E	238	0.12	2003	76
*dhps*581G	124	0.00	2001	73

Figure A1.1 Summary of *dhps* marker data. Left hand panels: Data locations for *dhps*437G (top), *dhps*540E (middle) and *dhps*581G (bottom) used in the *dhps*540E model. The colour of the data points represented the observed prevalence of the marker and the size of the circle is proportional to the sample size of the study. Right hand panels: The number of surveys conducted for *dhps*437G (top), *dhps*540E (middle) and *dhps*581G (bottom) over the time period 1987-2008.

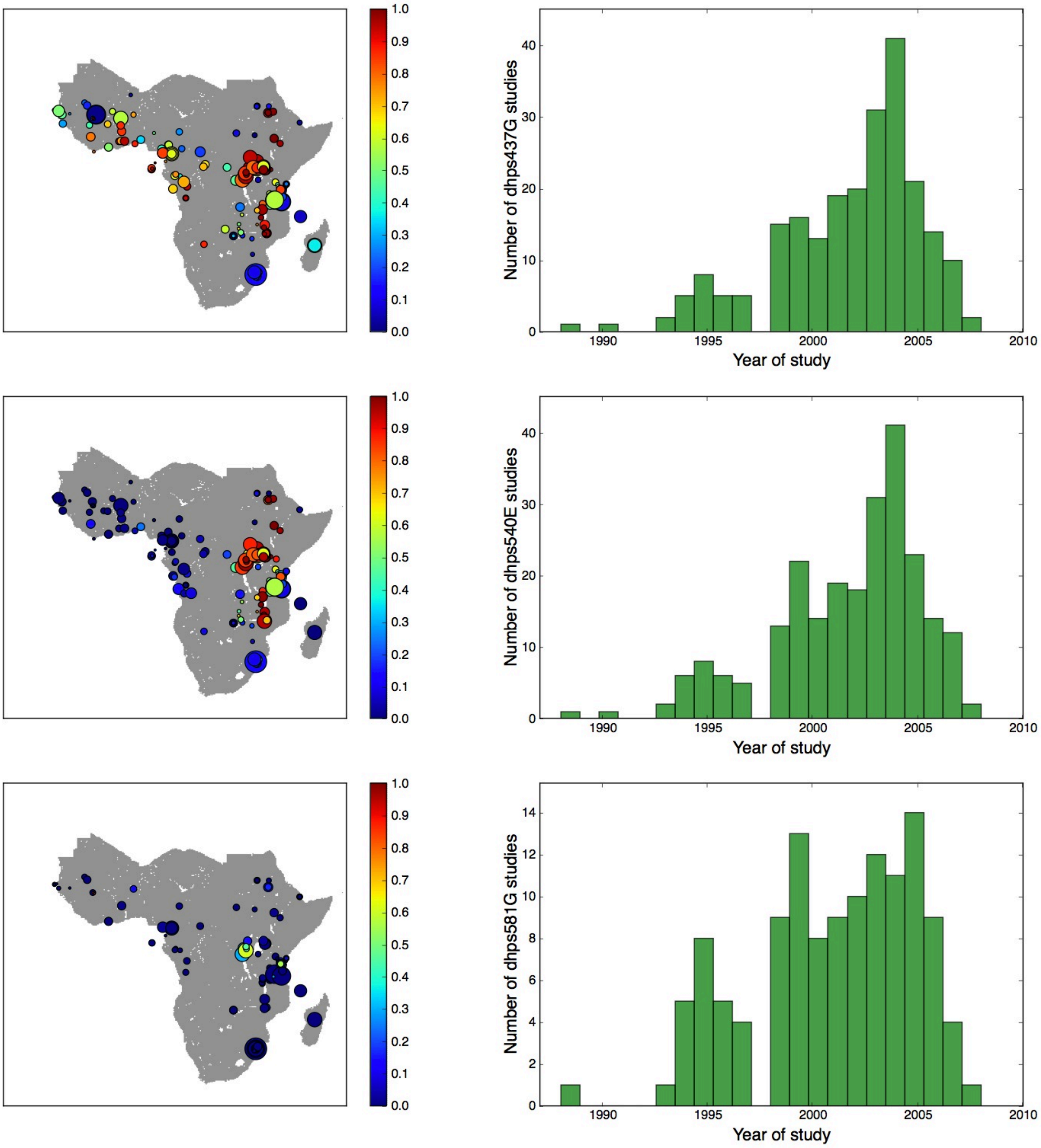
1.0
0.9
0.8
0.7
0.6
0.5
0.4
0.3
0.2
0.1
0.0
Number of dhps437G studies
Number of dhps540E studies
Number of dhps581G studies
Year of study
1990
1995
2000
2005
2010
0
10
20
30
40
2
4
6
8
10
12
14
