# Supplementary material for: Spatiotemporal mathematical modelling of mutations of the dhps gene in African Plasmodium falciparum
Source: Malar J. 2013 Jul 17;12:249. doi: 10.1186/1475-2875-12-249 (PMC3728261; doi:10.1186/1475-2875-12-249)

Additional file 2. Model based geostatistical framework for generating maps of *dhps*540E prevalence

The full details of the methodology for the spatio-temporal prediction of the *dhps*540E molecular marker prevalence are presented here. The model was developed using a model-based geostatistics (MBG) framework [1] and the parameters were estimated using Bayesian inference and Markov Chain Monte Carlo (MCMC) simulation [2].

Employing a Bayesian MBG approach offers several advantages. The classical geostatistical framework allows for spatial prediction [1, 3], while the generalized linear modelling permits flexibility in the response variable being modelled [4]. The Bayesian nature of the methodology allows parameter estimation and quantification of the uncertainty [3, 5].

A2.1 Geostatistical model

Joint probability model for *dhps*540E markers

A schematic representation of the full model is given in Figure A2.1. The number of individuals in the ith study, conducted at location $\underline{x}_i$ in year t_i, that were positive for the *dhps*540E marker (N_i^+) was assumed to be binomially distributed, given the number of individuals tested in the ith study (N_i) and the probability $P(\underline{x}_i,t_i)$:

$$N_i^+ \big| N_i, P(\underline{x}_i,t_i) \sim Binomial(N_i, P(\underline{x}_i,t_i)) .$$

The probability, $P(\underline{x},t)$, at an arbitrary location $\underline{x}$ and time t, was modeled as the inverse logit transformation of the sum of a random field, $f(\underline{x},t)$, and an unstructured random component, $\varepsilon(\underline{x},t)$:

$$P(\underline{x},t) = \text{logit}^{-1}(f(\underline{x},t) + \varepsilon(\underline{x},t)) .$$

The unstructured components, $\varepsilon(\underline{x},t)$, were assumed to be independent and identically distributed with zero mean and variance V

$$\varepsilon(\underline{x},t)\big|V \sim N(0,V).$$

The random field, $f(\underline{x},t)$, was modeled as a stationary Gaussian process, with mean function $\mu(\underline{x},t)$ and covariance function $C(\underline{x},t)$:

$$f(\underline{x},t)\big|\underline{\theta}_M,\underline{\theta}_C \sim GP(\mu(\underline{x},t),C(\underline{x},t)),$$

where $\underline{\theta}_M$ and $\underline{\theta}_C$ are vectors of parameters that specify the mean and covariance function, respectively. Specifically, it was assumed that the mean function varies linearly in time and with malaria transmission intensity

$$\mu(\underline{x},t)=\beta_0+\beta_1 t+\beta_2 m(\underline{x},t), \qquad (1)$$

where $\underline{\theta}_M=\{\beta_0,\beta_1,\beta_2\}$. The covariance function was chosen to be a version of the spatio-temporal structure advocated by Stein [6] and adopted previously by Hay *et al.* [7] and Gething *et al.* [8]. The covariance between a study conducted at location $\underline{x}_i$ in year t_i, and a study performed at $\underline{x}_j$ in year t_j was given by

$$C(\underline{x}_i,t_i,\underline{x}_j,t_j)=\sigma^2\gamma(0)\frac{\Delta x^{\gamma(\Delta t)}\kappa_{\gamma(\Delta t)}(\Delta x)}{2^{\gamma(\Delta t)-1}\Gamma(\gamma(\Delta t)+1)} \qquad (2)$$

where Γ is the gamma function, κ_γ is the modified Bessel function of the second kind of order γ, $\Delta t=\left|t_i-t_j\right|$ and

$$\gamma(\Delta t)=(2\rho+2(1-\rho)e^{-\Delta t/\phi_t})^{-1}.$$

The distance Δx was given by

$$\Delta x = \frac{2\sqrt{\gamma(\Delta t)}D_{GC}(\underline{x}_i, \underline{x}_j)}{\phi_x}$$

where $D_{GC}(\underline{x}_i, \underline{x}_j)$ is the great circle distance between locations $\underline{x}_i$ and $\underline{x}_j$. In the notation adopted here, the parameter ϕ_t refers to the temporal scale factor, ρ to the temporal limiting correlation, σ to the partial sill and ϕ_x to the spatial range. The covariance parameters were: $\underline{\theta}_C = \{\phi_t, \rho, \sigma, \phi_x\}$.

The joint probability model for the n *dhps*540E observations, the structured and unstructured components, given the model parameters, the space-time location of the data and the sample sizes was therefore given by

$$p(\underline{N}^+, f, \underline{\varepsilon} \mid \underline{N}, \underline{X}, \underline{t}, \underline{\theta}_M, \underline{\theta}_C, V) = \prod_{i=1}^{n} p(N_i^+ \mid f(\underline{x}_i, t_i), \varepsilon(\underline{x}_i, t_i), N_i, \underline{x}_i, t_i) p(\varepsilon(\underline{x}_i, t_i) \mid V) \cdot p(f(\underline{X}, \underline{t}) \mid \underline{\theta}_M, \underline{\theta}_C, \underline{X}, \underline{t})$$

where $\underline{N}^+, \underline{N}, \underline{X}$ and $\underline{t}$ are the augmented set of positive *dhps*540E responses, number of samples tested, location and time of the study, respectively, for the set of studies. That is, for example,

$$\underline{N}^+ = [N_1^+, N_2^+, \ldots, N_i^+, \ldots, N_n^+]$$
$$\underline{X} = [\underline{x}_1, \underline{x}_2, \ldots, \underline{x}_i, \ldots, \underline{x}_n]$$

Inclusion of the *dhps*437G and *dhps*581G markers

To incorporate the information contained within the *dhps*437G and *dhps*581G marker data into the model framework outlined in the previous section, factor potentials were introduced that multiply the joint probability model [9, 10]. Essentially, the presence of *dhps*437G or *dhps*581G data at a location without *dhps*540E data placed an upper (*dhps*437G) or lower (*dhps*581G) constraint on the predictive *dhps*540E prevalence allowed by the model.

For a space-time location $(\underline{x}_k, t_k)$ that was not associated with a *dhps*540E observation, but was associated with *dhps*437G data (N^+_{437} of the N_{437} samples are positive for the *dhps*437G marker), the likelihood, given the random field and unstructured random component was modified:

$$I(N^+_{437}, N_{437}) \prod_i^n p(N^+_i \,|\, f(\underline{x}_i, t_i), \varepsilon(\underline{x}_i, t_i), N_i, \underline{x}_i, t_i)$$

where $I(N^+_{437}, N_{437})$ is an indicator function such that

$$I(N^+_{437}, N_{437}) = \begin{cases} 1, & \text{if } P(\underline{x}_k, t_k) \leq N^+_{437} / N_{437} \\ 0, & \text{otherwise} \end{cases}$$

where $P(\underline{x}_k, t_k) = \text{logit}^{-1}(f(\underline{x}_k, t_k) + \varepsilon(\underline{x}_k, t_k))$ is the predicted model prevalence of the *dhps*540E marker at location $\underline{x}_k$ and time t_k. A separate factor potential (and hence indicator function) was used for each space-time location where *dhps*437G data was available but *dhps*540E data was not.

In a similar fashion, *dhps*581G data (N^+_{581}, N_{581}) at a space-time location $(\underline{x}_k, t_k)$ was incorporated into the model by multiplying the likelihood by the indicator function

$$I(N^+_{581}, N_{581}) = \begin{cases} 1, & \text{if } P(\underline{x}_k, t_k) \geq N^+_{581} / N_{581} \\ 0, & \text{otherwise} \end{cases}$$

Prior specification

Priors were specified for the mean and covariance parameters $\{\underline{\theta}_M, \underline{\theta}_C, V\} = \{\beta_0, \beta_1, \beta_2, \phi_t, \rho, \sigma, \phi_x, V\}$. The logarithm of the partial sill (σ) and the spatial range (ϕ_x) were assigned skew-normal priors:

$$\log(\sigma) \,|\, \mu_\sigma, V_\sigma, \alpha_\sigma \sim \text{SkewNormal}(\mu_\sigma, V_\sigma, \alpha_\sigma),$$
$$\log(\phi_x) \,|\, \mu_\phi, V_\phi, \alpha_\phi \sim \text{SkewNormal}(\mu_\phi, V_\phi, \alpha_\phi).$$

The temporal scale (ϕ_t) was given a relatively vague prior

$$\phi_t \sim \text{Exponential}(0.1).$$

The temporal limiting correlation (ρ) was assigned a uniform prior

$$\rho \sim \text{Uniform}(0,1).$$

Noninformative priors were specified for the regression coefficients in the mean function of the Gaussian process

$$p(\beta_0, \beta_1, \beta_2) \propto 1 .$$

The inverse of the variance of the unstructured random component ($1/V$) was assigned a diffuse Gamma prior with mean 0.25

$$V^{-1} \sim \text{Gamma}(0.001, 0.004).$$

A2.2 Implementation

The implementation of the model proceeds with two main steps: inference and prediction, as detailed below.

Parameters estimation (inference stage)

In the parameter estimation stage, the output of the MBG model was the posterior probability distribution of the model parameters, given the observed data. Samples were drawn from the posterior distribution of the model mean and covariance parameters ($\underline{\theta}_M, \underline{\theta}_C, V$) and the random field ($f(\underline{x}_i, t_i)$) at each location where *dhps*540E, 437G or 581G data was available, using a MCMC approach. The MCMC algorithm was implemented in the Python [11] package PyMC [12]. PyMC is an open-source Python module that implements Bayesian statistical models and fitting algorithms, including MCMC.

The mean and covariance parameters ($\underline{\theta}_M, \underline{\theta}_C, V$) were updated jointly within the MCMC algorithm using Metropolis steps while the values of the space-time random field at the data locations and times were updated using Gibbs steps. The unstructured random components ($\varepsilon(\underline{x}_i, t_i)$) were updated separately using Metropolis steps.

Spatio-temporal mapping (prediction stage)

In the prediction stage, the output was the posterior distribution of the prevalence of the *dhps*540E marker at each space-time point of predictive interest (here each location on a 25 x 25 km grid in sub-Saharan Africa from 1990-2010). From the output of the inference stage, parameter values were available for the jth sample $\{\beta_0^j, \beta_1^j, \beta_2^j, \phi_t^j, \rho^j, \sigma^j, \phi_x^j, V^j\}$, $j = 1,...,m$ and for $f^j(\underline{x}_i, t_i)$, $j = 1,...,m$ for each of the data locations ($i = 1,...,n$). Here, the number of data locations (n) was the number of locations where either *dhps*540E, 437G or 581G data was available.

To generate a predictive map for 2010, for each of the samples ($j = 1,...,m$), for each of the prediction locations on a 25 x 25 km grid ($k = 1,...,T$) of sub-Saharan Africa, the conditional distribution of the random field, $f^j(\underline{x}_k, 2010)$, was sampled from a multivariable Normal distribution with mean and covariance matrix given, respectively, by

$$\mu^j(\underline{x}_k, 2010) + C^j(\underline{X}, \underline{t}, \underline{x}_k, 2010)^T C^j(\underline{X}, \underline{t}, \underline{X}, \underline{t})^{-1}(f^j(\underline{X}, \underline{t}) - \mu^j(\underline{X}, \underline{t})) \text{ and}$$
$$C^j(\underline{x}_k, 2010, \underline{x}_k, 2010) - C^j(\underline{X}, \underline{t}, \underline{x}_k, 2010)^T C^j(\underline{X}, \underline{t}, \underline{X}, \underline{t})^{-1} C^j(\underline{X}, \underline{t}, \underline{x}_k, 2010),$$

where $\mu^j(\underline{x}_k, 2010)$ and $C^j(\underline{x}_k, 2010, \underline{x}_k, 2010)$ are scalar quantities of the mean function value (Eq 1) and the covariance function value (Eq 2) in 2010 at $\underline{x}_k$, respectively, $f^j(\underline{X}, \underline{t})$, $\mu^j(\underline{X}, \underline{t})$ and $C^j(\underline{X}, \underline{t}, \underline{x}_k, 2010)$ are vectors of length n of the random field, mean function and covariance function values at the n data locations:

$$\mu^j(\underline{X}, \underline{t}) = [\mu^j(\underline{x}_1, t_1), ..., \mu^j(\underline{x}_n, t_n)], \; f^j(\underline{X}, \underline{t}) = [f^j(\underline{x}_1, t_1), ..., f^j(\underline{x}_n, t_n)],$$
$$C^j(\underline{X}, \underline{t}, \underline{x}_k, 2010) = [C^j(\underline{x}_1, t_1, \underline{x}_k, 2010), ..., C^j(\underline{x}_n, t_n, \underline{x}_k, 2010)]$$

and $C^j(\underline{X},\underline{t},\underline{X},\underline{t})$ is an n by n matrix with elements

$C^j(\underline{X},\underline{t},\underline{X},\underline{t})_{r,p} = C^j(\underline{x}_r,t_r\underline{x}_p,t_p)_{r,p}$. The subscript j denotes that the quantity was evaluated with the jth sample from the posterior distribution.

To this $f^j(\underline{x}_k,2010)$ sample, the unstructured component (drawn from $\varepsilon^j(\underline{x}_k,2010) \sim Normal(0,V^j)$) was added. Finally, applying an inverse logit transformation gave the jth sample from the posterior distribution for the *dhps*540E prevalence at location $\underline{x}_k$ in 2010. Repeating this for each of the m samples formed the set of *dhps*540E prevalence samples at $\underline{x}_k$ in 2010:

$$\{p^j(\underline{x}_k,2010),\ j = 1,...,m\}.$$

The point estimate of *dhps*540E at $\underline{x}_k$ in 2010 was defined as the median of this set. Repeating for each prediction location on a 25 x 25 km grid resulted in a median map of *dhps*540E prevalence in 2010. The predictive procedure can be repeated for any year of interest. An associated uncertainty map accompanies the median maps presented in the main text. The uncertainty at each prediction location was the sample standard deviation from the set $\{p^j(\underline{x}_k,2010),\ j = 1,...,m\}$. Note that the higher the standard deviation, the higher the uncertainty in the distribution of the prevalence of *dhps*540E.

A2.3 Transmission intensity

The Malaria Atlas Project (MAP) has developed spatio-temporal MBG frameworks to generate world *P. falciparum* endemicity maps, the most recent of which was created for 2010 [8]. The computational demands to generate predictive *P. falciparum* maps at each year from 1990-2010 are substantial under the current MAP framework. In

this model, only the spatial predictions for 2010 were included in the mean function of the Gaussian process. That is;

$$\mu(\underline{x},t) = \beta_0 + \beta_1 t + \beta_2 m(\underline{x},2010)$$

In this way, the *P. falciparum* transmission was incorporated into the model as a mechanism for how the prevalence of *dhps*540E changes spatially.

References

1. Diggle PJ, Tawn J, Moyeed R: **Model based geostatistics**. *J R Stat Soc Ser C Appl Stat* 1998, **47**:299-350.
2. Gilks WR, Richardson S, Spiegelhalter DJ: **Markov chain Monte Carlo in practice**: Chapman & Hall/CRC; 1996.
3. Diggle PJ, Ribeiro PJ: **Model-based geostatistics**: Springer; 2007.
4. McCullagh P, Nelder JA: **Generalized linear models**: Chapman & Hall/CRC; 1989.
5. Gelman A, Carlin JB, Stern HS, Rubin DB: **Bayesian data analysis**: CRC press; 2004.
6. Stein M: **Space-time covariance functions**. *J Am Stat Assoc* 2005, **100**:310-321.
7. Hay S, Guerra C, Gething P, Patil A, Tatem A, Noor A, Kabaria C, Manh B, Elyazar I, Brooker S *et al*: **A world malaria map: *Plasmodium falciparum* endemicity in 2007**. *PLoS Med* 2009, **6**:e1000048.
8. Gething PW, Patil AP, Smith DL, Guerra CA, Elyazar IRF, Johnston GL, Tatem AJ, Hay SI: **A new world malaria map: *Plasmodium falciparum* endemicity in 2010**. *Malar J* 2011, **10**:378.
9. Christakos G: **On the assimilation of uncertain physical knowledge bases: Bayesian and non-Bayesian techniques**. *Adv Water Resour* 2002, **25**:1257-1274.
10. Lauritzen SL, Dawid AP, Larsen BN, Leimer HG: **Independence properties of directed Markov fields**. *Networks* 1990, **20**:491-505.
11. **Python programming language** [http://www.python.org]
12. Patil A, Huard D, Fonnesbeck C: **PyMC: Bayesian stochastic modelling in Python**. *J Stat Softw* 2010, **35**:1-81.

Figure A2.1. Representation of the *dhps*540E probability model in a directed acyclic graph. Here arrows indicate conditional dependencies. The brown, grey, green and blue ovals represent model parameters, data input (variables that have been observed), covariates and predictive surfaces, respectively. The *dhps*437G and the *dhps*581G constaints are represented by the grey rectangles.

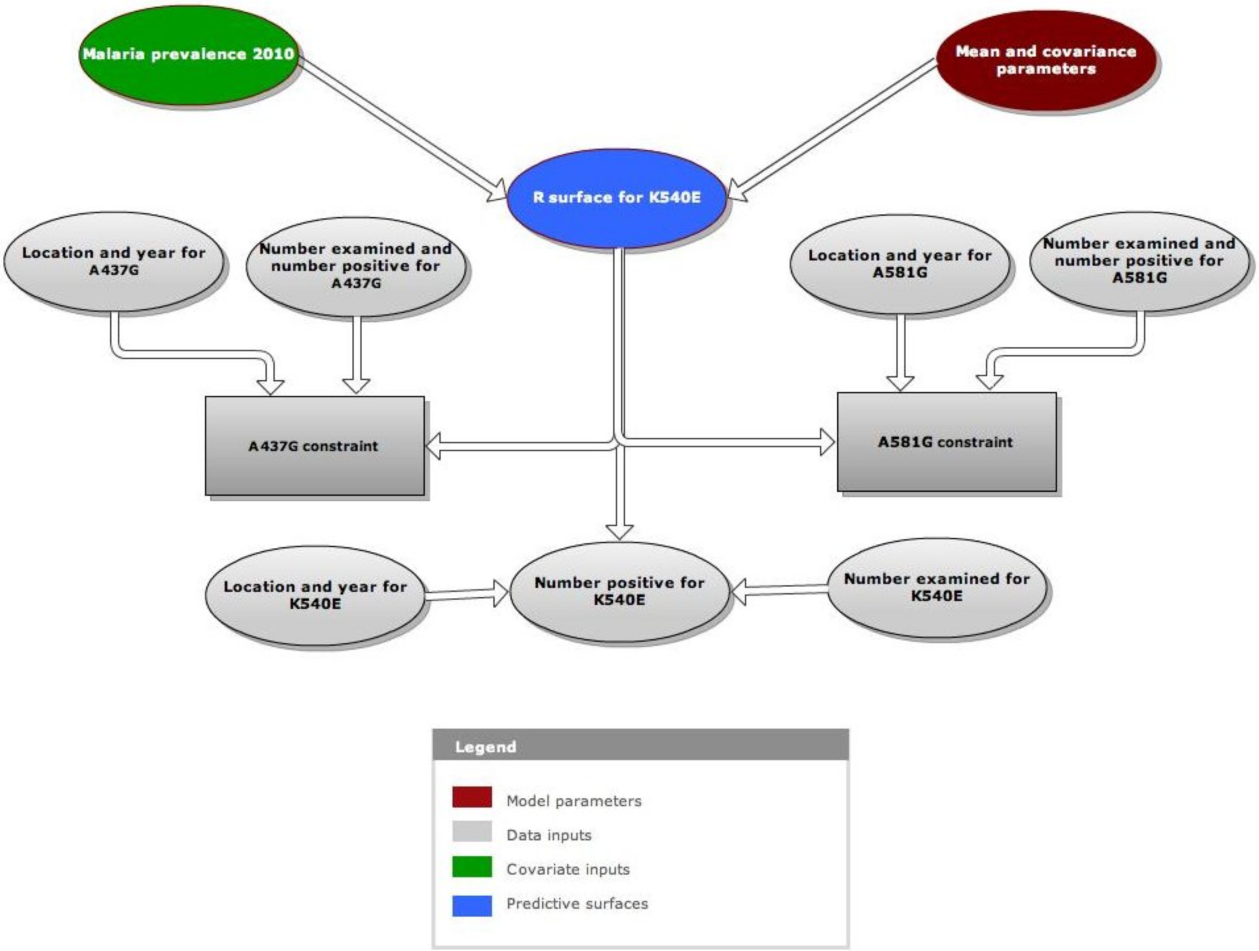

Supplement: Additional file 2 — Model based geostatistical framework for generating maps of dhps540E prevalence. [file 1475-2875-12-249-S2.pdf]
