# Supplementary material for: Spatiotemporal mathematical modelling of mutations of the dhps gene in African Plasmodium falciparum
Source: Malar J. 2013 Jul 17;12:249. doi: 10.1186/1475-2875-12-249 (PMC3728261; doi:10.1186/1475-2875-12-249)

Additional file 3. Model validation procedures and results

The model validity was assessed to ensure that the interpretation of the model output was valid. The *dhps*540E dataset was divided into five groups, at random, each of which was treated as a validation set to test the model's predictive ability. For each of the five subsets of data, the model was run with the dataset withheld and the predictive ability of the model was tested against the withheld data. The predictive results for each of the five subsets of data were pooled, so that each *dhps*540E observation had an associated predictive validation value, from a model where this observation was not included.

Since the validation procedures were carried out on validation datasets that had data withheld, they do not measure the ability of the model fitted to the entire *dhps*540E dataset to predict *dhps*540E prevalence. As expected, the full model's predictive ability of *dhps*540E prevalence is very good (correlation coefficient > 0.97).

A3.1 Validation procedures

The median of the predictive posterior distribution at each of the data locations was used to compare to the observed prevalences to calculate the correlation coefficient, the mean error and the mean absolute error. The predictive ability was assessed by comparing the observed prevalence ($P_A(\underline{x}_i,t_i)$) at location $(\underline{x}_i,t_i)$ to the associated validation value $P_V(\underline{x}_i,t_i)$, $i=1,\ldots,n$, using:

1. The correlation coefficient between $P_A(\underline{x}_i,t_i)$ and $P_V(\underline{x}_i,t_i)$ as a measure of linear association.
2. The mean prediction error (ME) as a measure of bias

$$\text{ME} = \frac{1}{n}\sum_{i=1}^{n} P_V(\underline{x}_i,t_i) - P_A(\underline{x}_i,t_i).$$

3. The mean absolute error (MAE) as a measure of the average accuracy of individual predictions

$$\text{MAE} = \frac{1}{n}\sum_{i=1}^{n}\left|P_V(\underline{x}_i, t_i) - P_A(\underline{x}_i, t_i)\right|.$$

A3.2 Validation results

The mean error in the generation of *dhps*540E prevalence estimates at the 238 data locations was 1.83%, with values in units of prevalence on the percentage scale. This indicated that overall bias was small in that, on average, the model predictions overestimated the observed prevalence by less than 2%. The mean absolute error was found to be 12.9%, in units of prevalence on the percentage scale, illustrating that the average magnitude of the prediction error was just under 13%. The correlation coefficient between the observed *dhps*540E prevalences and their associated validation values was 0.86, demonstrating a very strong linear agreement between the observed and predicted prevalences. This strong association is supported by the scatter plot shown in Figure A3.1(a), which shows the observed *dhps*540E prevalence (x-axis) versus the predicted (validation) values (y-axis).

Figure A3.1(b) shows a probability-probability plot of the fraction of observations (y-axis) that fell within a predictive credible interval of a given size (x-axis). The predictive credible intervals for each observation were created from the posterior samples. The figure shows that reliability of the credible intervals was reasonably strong, especially for narrower credible intervals.

Figure A3.1. Model validation plots. (a) Scatter plot of the observed *dhps*540E prevalence (x-axis) versus the predicted (validation) values (y-axis). The solid blue line shows a 1:1 reference line. (b) Probability-probability plot of the fraction of observations (y-axis) that fell within a predictive credible interval of a given size (x-axis). The dashed blue line shows a 1:1 reference line.

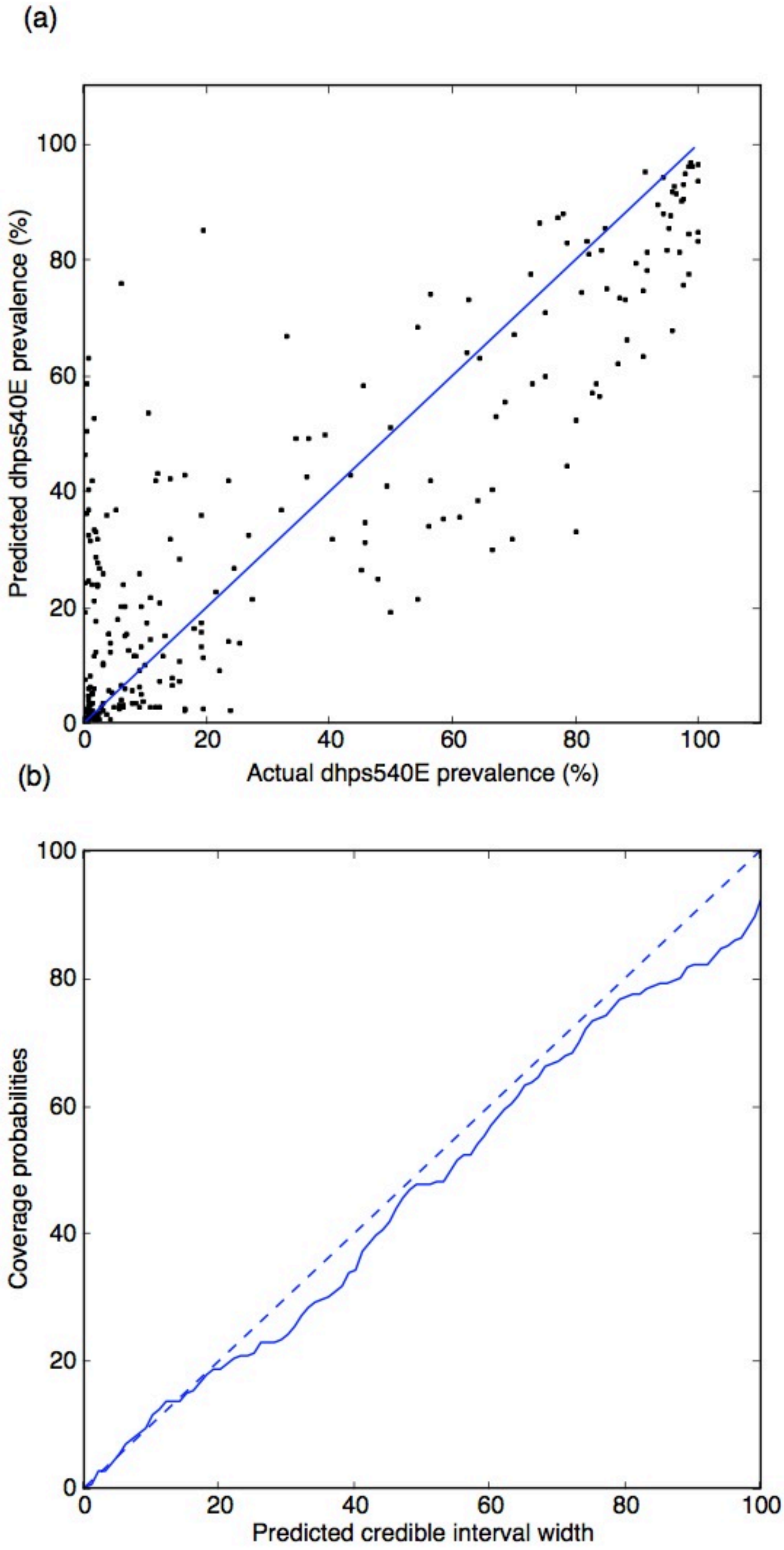

Supplement: Additional file 3 — Model validation procedures and results. [file 1475-2875-12-249-S3.pdf]
